# Opaganib Protects against Radiation Toxicity: Implications for Homeland Security and Antitumor Radiotherapy

**DOI:** 10.3390/ijms232113191

**Published:** 2022-10-29

**Authors:** Lynn W. Maines, Randy S. Schrecengost, Yan Zhuang, Staci N. Keller, Ryan A. Smith, Cecelia L. Green, Charles D. Smith

**Affiliations:** Apogee Biotechnology Corporation, 1214 Research Blvd, Suite 2015, Hummelstown, PA 17036, USA

**Keywords:** opaganib, ABC294640, sphingolipid, sphingosine kinase, radiation, xenograft

## Abstract

Exposure to ionizing radiation (IR) is a lingering threat from accidental or terroristic nuclear events, but is also widely used in cancer therapy. In both cases, host inflammatory responses to IR damage normal tissue causing morbidity and possibly mortality to the victim/patient. Opaganib, a first-in-class inhibitor of sphingolipid metabolism, has broad anti-inflammatory and anticancer activity. Opaganib elevates ceramide and reduces sphingosine 1-phosphate (S1P) in cells, conditions that increase the antitumor efficacy of radiation while concomitantly suppressing inflammatory damage to normal tissue. Therefore, opaganib may suppress toxicity from unintended IR exposure and improve patient response to chemoradiation. To test these hypotheses, we first examined the effects of opaganib on the toxicity and antitumor activity of radiation in mice exposed to total body irradiation (TBI) or IR with partial bone marrow shielding. Oral treatment with opaganib 2 h before TBI shifted the LD_75_ from 9.5 Gy to 11.5 Gy, and provided substantial protection against gastrointestinal damage associated with suppression of radiation-induced elevations of S1P and TNFα in the small intestines. In the partially shielded model, opaganib provided dose-dependent survival advantages when administered 4 h before or 24 h after radiation exposure, and was particularly effective when given both prior to and following radiation. Relevant to cancer radiotherapy, opaganib decreased the sensitivity of IEC6 (non-transformed mouse intestinal epithelial) cells to radiation, while sensitizing PAN02 cells to in vitro radiation. Next, the in vivo effects of opaganib in combination with radiation were examined in a syngeneic tumor model consisting of C57BL/6 mice bearing xenografts of PAN02 pancreatic cancer cells and a cross-species xenograft model consisting of nude mice bearing xenografts of human FaDu cells. Mice were treated with opaganib and/or IR (plus cisplatin in the case of FaDu tumors). In both tumor models, the optimal suppression of tumor growth was attained by the combination of opaganib with IR (± cisplatin). Overall, opaganib substantially protects normal tissue from radiation damage that may occur through unintended exposure or cancer radiotherapy.

## 1. Introduction

Pathologic inflammation is a pronounced consequence from ionizing radiation (IR) [1,2,3,4] that can occur through unintended exposure, e.g., a nuclear accident or terroristic event, or intended exposure, i.e., cancer radiotherapy. Radiation-induced production of proinflammatory cytokines, particularly IL-1β, TNFα and IL-6, drives acute and chronic sequelae in the blood, peripheral lymphoid tissues, gastrointestinal (GI) tract and lungs [5,6,7]. In particular, radiation enteritis, aka GI-Acute Radiation Syndrome (GI-ARS), can be a serious complication from abdominal and pelvic radiation [8,9,10]. Mice exposed to IR of the abdomen experience GI-ARS that results in death approximately 10–16 days after exposure. Clinical and experimental studies of the acute and late effects of IR on normal and tumor cells have improved cancer radiotherapy schedules and modes of radiation delivery [11,12,13]. However, radiation therapy in cancer continues to damage surrounding normal tissue, and this frequently limits the amount of radiation that can be used for treatment as well as causing substantial morbidity and reduction in the patient’s quality of life. Bowel injuries that result in fistulas, strictures, and chronic malabsorption, as well as severe transmural fibrosis associated with inflammation, epithelial damage, and vascular sclerosis, are potentially life-threatening complications from abdominal radiotherapy [14,15]. Mitigation of radiation toxicity is also an area of concern for the Biomedical Advanced Research and Development Authority (BARDA) which seeks to develop medical countermeasures (MCMs) to treat the acute effects of high dose, whole-body radiation exposure and trauma to save lives in radiological or nuclear incidents (https://www.medicalcountermeasures.gov/barda/cbrn accessed on 1 October 2022).

The importance of sphingolipid metabolism in cancer and inflammatory diseases is being increasingly recognized (reviewed in [16,17,18,19]). Growth factors and inflammatory cytokines activate sphingomyelinases that hydrolyze sphingomyelin to form ceramide which induces apoptosis in tumor cells without disrupting quiescent normal cells. Additionally, ceramide can be further hydrolyzed by ceramidases to produce sphingosine, which is phosphorylated by sphingosine kinases (SK1 and SK2) to produce sphingosine 1-phosphate (S1P). S1P induces proliferation and protects against ceramide-induced apoptosis and is pro-inflammatory. Sphingolipids also regulate the sensitivities of tumor cells to anticancer drugs and IR (reviewed in [20,21,22]). For example, the ability of sphingosine to promote apoptosis in response to radiation treatment of prostate cancer cells was described by Nava et al. [23], and the role of the ceramide: S1P balance in regulating radiation sensitivity has been widely substantiated [24,25,26]. Therefore, disruption of metabolism of ceramide to S1P is a new method to enhance radiation sensitivity in tumor cells, while concurrently suppressing pathologic inflammation in adjacent tissues.

Opaganib (aka ABC294640) is an orally active, isozyme-selective inhibitor of SK2, and is competitive with respect to sphingosine [27,28]. Opaganib depletes S1P and elevates ceramide in tumor cells, suppresses signaling through pERK, pAKT and NFκB, and promotes autophagy and/or apoptosis [27,28,29,30,31]. Opaganib also down-regulates c-Myc in a variety of tumor cell lines [31,32,33,34]. Because it acts as a sphingosine mimetic, opaganib also inhibits dihydroceramide desaturase (DES1), increasing levels of dihydroceramides [32] and promoting autophagy in those cells. Opaganib has antitumor activity in a wide range of mouse models [27,31,32,33,35,36,37,38,39], as well as anti-inflammatory activity in several rodent models [40,41,42,43,44]. A phase I clinical trial with opaganib administered to patients with advanced solid tumors demonstrated that it is well-tolerated even with treatment of >40 weeks, and provided disease stabilization in most patients [45]. Opaganib is currently in phase II clinical testing in patients with cholangiocarcinoma (NCT03377179) or prostate cancer (NCT04207255). Because of its anti-inflammatory and antiviral properties, opaganib was evaluated in hospitalized patients with COVID-19 pneumonia [46], including a Phase 2a study that demonstrated that patients receiving oral opaganib required less supplemental oxygen and achieved earlier hospital discharge [47]. Subsequently, a Phase 2/3 multinational randomized, placebo-controlled study enrolled 475 adult subjects hospitalized with severe COVID-19 which demonstrated the safety of opaganib for these patients and a clinical benefit to patients requiring lower oxygen supplementation (62% reduction in rate of ventilation and death).

Specifically related to GI-ARS, we have demonstrated that activation of NFκB by TNFα is dose-dependently suppressed by opaganib, and that TNFα-induction of adhesion proteins involved in leukocyte recruitment and production of PGE_2_ are strongly suppressed by opaganib [44]. In models of inflammatory bowel disease, the effects of opaganib are associated with decreased levels of TNFα, IL-1β, IFN-γ and IL-6 and reduction of S1P levels in the colon [42,43]. We now hypothesize that opaganib will provide protection against IR toxicity in vivo, and may enhance the antitumor effects of radiation. Therefore, in the present studies, we examined the ability of opaganib to suppress IR-induced GI damage and mortality using total body irradiation (TBI) and partially shielded exposure models. Additionally, studies on the antitumor efficacy of opaganib in combination with IR are described herein.

## 2. Results

### 2.1. Protection against Radiation Toxicity Studies 

**Figure 1:**
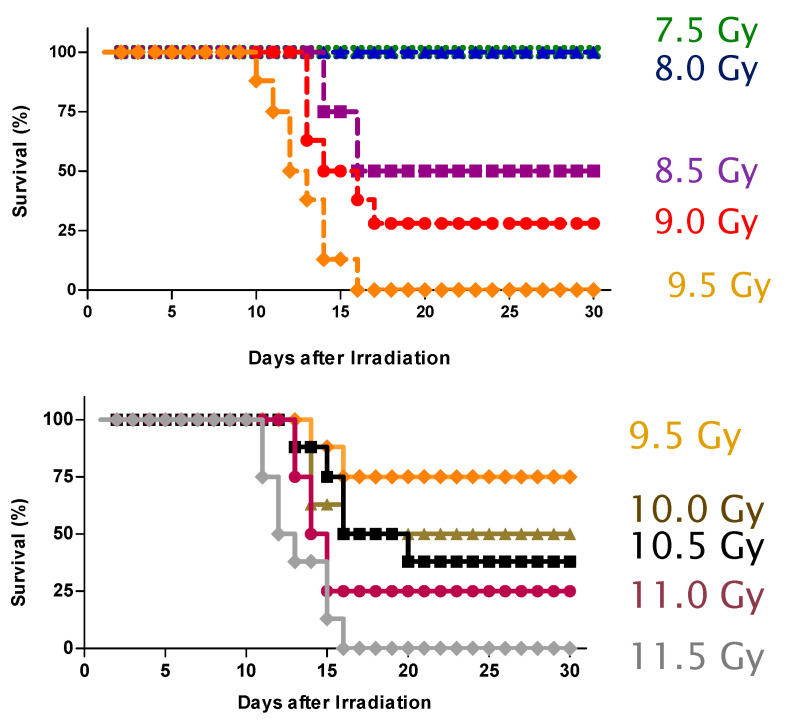
Opaganib protects against lethality from total body irradiation. Mice in the vehicle groups were exposed to 7.5, 8.0, 8.5, 9.0 or 9.5 Gy TBI (**Top Panel**), and mice in the opaganib-treated groups (100 mg/kg oral, 1 h prior to radiation) were exposed to 9.5, 10.0, 10.5, 11.0 or 11.5 Gy TBI (**Bottom Panel**). Survival was monitored for 30 days.

**Effect of opaganib on the lethality of TBI in C57BL/6 mice.** We have previously shown that opaganib inhibits deleterious GI inflammation in models of ulcerative colitis and Crohn’s Disease, and herein sought to determine if opaganib provides similar protection in mice exposed to lethal levels of radiation. For the initial studies, male C57BL/6 mice were pretreated orally with either vehicle (0.375% Tween-80) or 100 mg/kg opaganib 2 h before (−2 h) exposure to 9.5 Gy of TBI. Because death from GI and hematologic damage occurs within two weeks after exposure to radiation, animals were monitored for toxicity for the next 30 days and euthanized if moribund. Vehicle-treated mice experienced body weight losses of 7–10% and had pronounced diarrhea, indicative of severe GI damage, and all animals had to be sacrificed within 14 days of radiation exposure. In contrast, significant protection was observed in the opaganib-treated group, in which 71% of the mice survived indefinitely (*p* = 0.0009). For determination of the dose-modification factor (DMF) for opaganib, mice in the vehicle group were irradiated with doses ranging from 7.5 to 9.5 Gy and mice treated orally with 100 mg/kg of opaganib at −2 h were irradiated with doses ranging from 9.5 to 11.5 Gy (Figure 1). The LD_75/30_ of the opaganib-treated animals was 11 Gy and the LD_75/30_ of the vehicle-treated animals was 9.0 Gy, yielding a DMF for opaganib of 1.22. Mortality typically occurred between Days 10–16 following radiation, which is consistent with GI-ARS rather than hematologic toxicity which occurs more rapidly or fibrotic organ damage which is more delayed. It is well established that intestinal inflammatory damage and subsequent fibrosis account for a significant component of the morbidity caused by moderate levels of TBI. Therefore, further experiments focused on the small intestines as the target tissue for radiation damage. 

**Figure 2:**
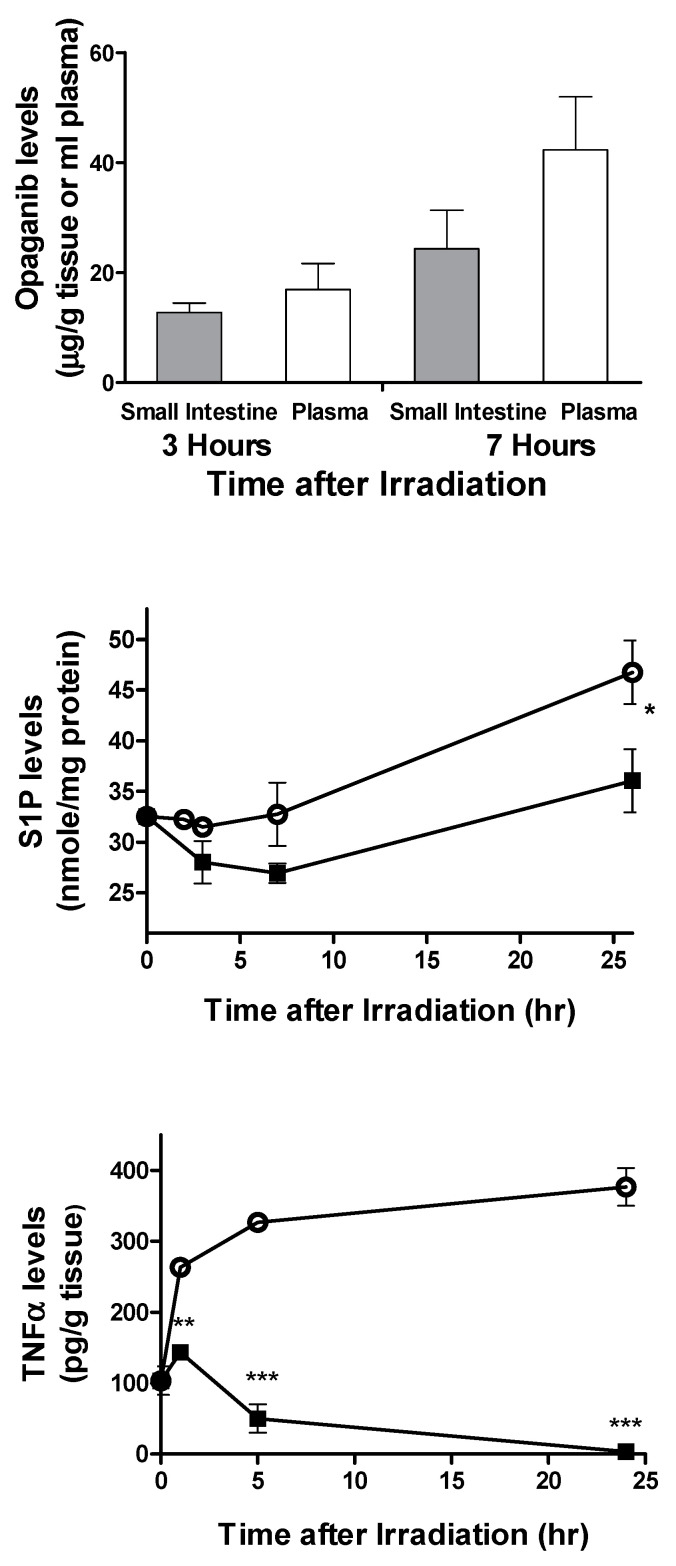
Biodistribution and pharmacodynamics of opaganib in the small intestine. **Top Panel**: Mice were treated with opaganib (100 mg/kg) orally and sacrificed at either 3 or 7 h. Plasma and extracts from the small intestines were analyzed for opaganib concentrations as described in the Materials and Methods section. **Middle and Bottom Panels**: Mice were treated with 0 (○) or 100 (∎) mg/kg opaganib orally 2 h before exposure to 9.5 Gy radiation and then sacrificed at the indicated times for evaluation of TNFα and S1P levels in the small intestine (*n* = 3–5 mice per time point; * *p* < 0.05, ** *p* < 0.01, *** *p* < 0.001).

**Accumulation and pharmacodynamics of opaganib in mouse small intestine.** To assess the delivery of opaganib to the target tissue, mice were treated orally with 100 mg/kg opaganib and then sacrificed at 3 or 7 h after treatment. The small intestines were harvested and analyzed for opaganib levels as described in the Materials and Methods section. As shown in Figure 2, opaganib concentrations in the small intestine were 33 μM at 3 h and 65 μM at 7 h after drug administration (calculated as 1.0 mL/g of tissue). These concentrations of opaganib were slightly lower than the concurrent concentrations in the plasma, indicating excellent delivery of the drug to the GI tissue. Because the K_i_ of opaganib for the inhibition of SK2 is 9.3 μM [28], opaganib reached therapeutic levels in the target tissue for an extended period of time. In parallel groups, mice were treated with 0 or 100 mg/kg opaganib 2 h before exposure to 9.5 Gy of TBI and sacrificed at times indicated in Figure 2. Small intestines were harvested and analyzed for S1P and TNFα levels by ELISAs. In vehicle-treated mice, S1P levels were substantially increased at 26 h after irradiation. In contrast, pretreatment with opaganib caused a transient decrease in S1P levels that returned to baseline at the 26 h time point (*p* < 0.05 compared with vehicle). In vehicle-treated mice, TNFα expression in the small intestines was up-regulated as early as 1 h after TBI and remained highly elevated for at least 26 h. In contrast, pretreatment with opaganib not only blocked the induction of TNFα by TBI but also reduced tissue TNFα levels below the baseline level. Therefore, oral administration of opaganib results in prolonged biodistribution of the drug into the small intestine at sufficient levels to inhibit SK2 and suppress radiation-induced inflammation. 

**Figure 3:**
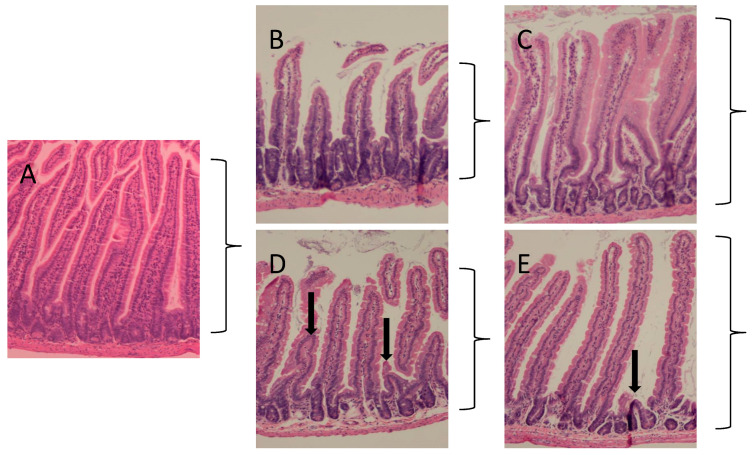
Effects of opaganib and radiation on the morphology of the small intestine. C57BL/6 mice were treated with 0 or 100 mg/kg opaganib orally 2 h before exposure to 9.5 Gy radiation. Control mice did not receive opaganib or radiation. Animals were then sacrificed on Day 4 or 10, and the small intestines were sectioned and stained with H&E. Representative sections are shown. Panel (**A**): unirradiated control, Panel (**B**): vehicle treated at 4 days, Panel (**C**): opaganib-treated at 4 days, Panel (**D**): vehicle treated at 10 days and Panel (**E**): opaganib-treated at 10 days. Villus height is indicated by the brackets and arrows indicate crypt destruction.

**Effects of opaganib on GI damage following TBI**. Mice were treated orally with 0 or 100 mg/kg opaganib 1 h prior to exposure to 9.5 Gy TBI. After 4 or 10 days, the animals were sacrificed and the small intestines were isolated, sectioned and stained with H&E. As shown in Figure 3, radiation exposure resulted in decreases in villus height (brackets) in the vehicle-treated animals at 4 (Panel B) and 10 (Panel D) days after radiation compared with non-irradiated controls (Panel A). In contrast, the villus heights were maintained in the opaganib-treated mice at Days 4 (Panel C) and 10 (Panel E). At Day 10, there is evidence of crypt destruction (arrows) in both vehicle- (Panel C) and opaganib-treated (Panel E) groups; however, it was more profound in the vehicle group. The numbers of cells/villi in the treatment groups were quantified and demonstrated significantly more cells present at 4 days after irradiation in opaganib-treated mice compared to vehicle controls (*** *p* < 0.001) with this difference between treatments nearly resolving by Day 10. 

**Figure 4:**
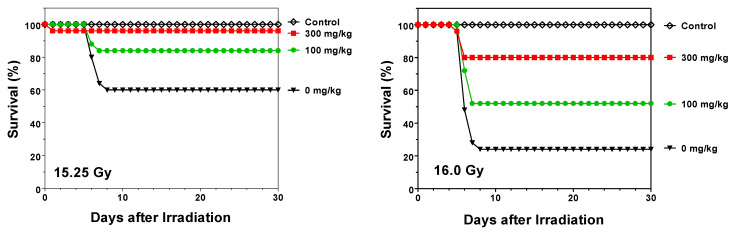
Single-dose opaganib improves survival of partially shielded irradiated mice. C57BL/6 mice were orally treated with 0 (▼), 100 (●) or 300 (∎) mg/kg opaganib 4 h before being exposed to 15.25 (**Left Panel**) or 16.0 (**Right Panel**) Gy irradiation with 5% bone marrow shielding. Control mice did not receive opaganib or radiation (◊). Mice were monitored for survival for 30 days.

**Effect of opaganib on the lethality of partially shielded irradiation in C57BL/6 mice.** Additional survival studies were conducted in a partially shielded (5% of bone marrow) model that more closely recapitulates the expected exposure pattern from unintended radiation exposure (accidental or terroristic) or exposure of normal tissues in cancer patients undergoing radiotherapy. Partial shielding of the bone marrow allows much higher doses of radiation to be delivered than in the TBI model, and this provides a GI-ARS model in which euthanasia is required between Days 5–8 because of loss of intestinal function. In the first series of studies, opaganib was administered as a single oral dose either before or after radiation exposure. As shown in Figure 4, vehicle-treated mice exposed to 15.25 Gy of radiation experienced a mortality rate of 40% between Days 5–8. Opaganib administered 4 h prior to irradiation at 15.25 Gy provided a survival advantage, with 100 and 300 mg/kg opaganib reducing mortality to 16% and 4%, respectively (*p* < 0.01 for 300 mg/kg). Vehicle-treated mice exposed to 16.0 Gy of radiation (Figure 4) demonstrated a mortality rate of 76%, and this was reduced to 48% and 20% by pretreatment with 100 or 300 mg/kg opaganib (*p* < 0.05 and *p* < 0.001, respectively). 

**Figure 5:**
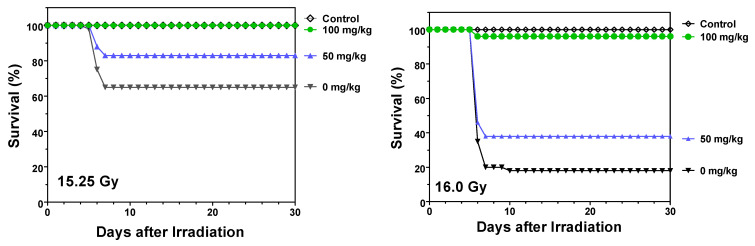
Pre- and post-radiation treatment with multiple-dose opaganib improves survival of partially shielded irradiated mice. C57BL/6 mice were orally treated with 0 (▼), 50 (▲) or 100 (●) mg/kg opaganib 4 h before being exposed to 15.25 (**Left Panel**) or 16.0 (**Right Panel**) Gy irradiation with 5% bone marrow shielding. Control mice did not receive opaganib or radiation (◊). Mice were treated with opaganib twice daily at the same dose as their initial treatment for a total of 3 days after radiation. Mice were monitored for survival for 30 days.

Because GI-ARS is a progressive injury culminating in organ failure after 5–8 days, further studies assessed the ability of opaganib to protect mice when given in multiple doses. Similar to the experiments described above, vehicle or opaganib (50 or 100 mg/kg) was administered to mice 4 h before (−4 h) exposure to either 15.25 or 16.0 Gy of radiation, and the mice were subsequently treated with the same dose of opaganib twice daily for a total of 3 days following radiation. As shown in Figure 5, vehicle-treated mice exhibited mortality rates of 35% and 82% after 15.25 and 16.0 Gy of radiation, respectively. Mortality from both radiation doses was substantially reduced in mice that received 50 mg/kg opaganib (*p* < 0.05 for 16.0 Gy) and was nearly eliminated in mice treated with 100 mg/kg opaganib, i.e., 0% and 4% mortality at 15.25 and 16.0 Gy (*p* < 0.01 and *p* < 0.001), respectively. 

**Figure 6:**
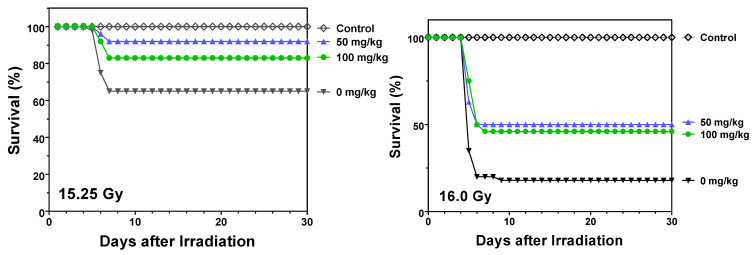
Post-radiation treatment with multiple-dose opaganib improves survival of partially shielded irradiated mice. C57BL/6 mice were orally treated with 0 (▼), 50 (▲) or 100 (●) mg/kg opaganib 24 h after being exposed to 15.25 (**Left Panel**) or 16.0 (**Right Panerl**) Gy irradiation with 5% bone marrow shielding. Control mice did not receive opaganib or radiation (◊). Mice were treated with opaganib twice daily at the same dose as their initial treatment for a total of 3 days after radiation. Mice were monitored for survival for 30 days.

Finally, multiple-dose experiments were also conducted in which the initial dose of opaganib was not administered until 24 h after radiation exposure. This model assesses the ability of opaganib to mitigate GI-ARS when immediate treatment is not be possible, e.g., in a mass-casualty scenario. As shown in Figure 6, multiple dose administration of opaganib started at 24 h after (+24 h) exposure to 15.25 of radiation decreased mortality from 39% for vehicle-treated mice to 8% and 18% for 50 and 100 mg/kg opaganib, respectively. Even at 16.0 Gy of radiation which resulted in 82% mortality for vehicle-treated mice, opaganib treatments of 50 or 100 mg/kg started 24 h after radiation decreased mortality to 56–58% (*p* < 0.01 for 50 mg/kg and *p* < 0.001 for 100 mg/kg). Opaganib (100 mg/kg) also provided significant protection (32% decrease in mortality) when started at 4 h after (+4 h) radiation (*p* < 0.05). 

**Figure 7:**
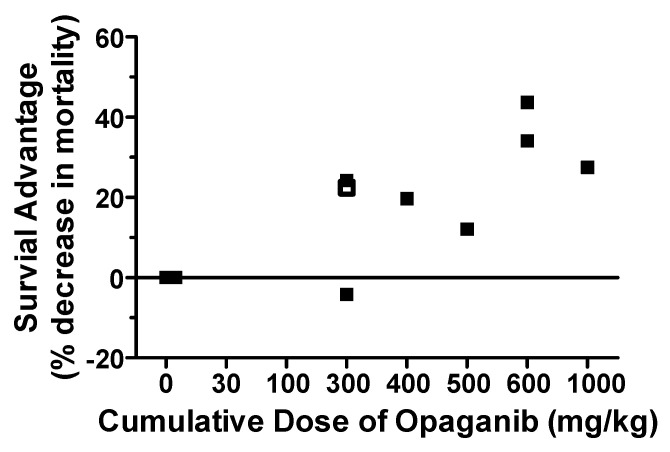
Survival advantage of opaganib when given after radiation exposure. The percent decrease in mortality observed at the indicated cumulative dose of opaganib given 24 h after 16.0 Gy of radiation across multiple experiments are plotted. A single dose of opaganib was given in the experiment indicated by the open symbol (□), whereas all other data derives from multiple dose treatment given over 2, 3 or 5 days following radiation (∎).

The effects of the cumulative dose of opaganib initiated 24 h after (+24 h) 16.0 Gy of radiation are plotted in Figure 7 to allow comparison across different treatment doses and durations. The combined data show that opaganib provides a dose-dependent Survival Advantage mitigating the lethality of this high dose of radiation. Statistical analyses demonstrated that the trend in risk-reduction for increasing opaganib cumulative dose was strong (HR = 0.82 for a 250 mg/kg increase in cumulative dose, 95% CI = 0.71 to 0.95; *p* = 0.0074). Specifically, a Survival Advantage was provided by opaganib cumulative doses as low as 300 mg/kg, and there was a trend toward greater efficacy with higher cumulative dose to at least 1000 mg/kg.

### 2.2. Cancer Radiotherapy Studies

**Figure 8:**
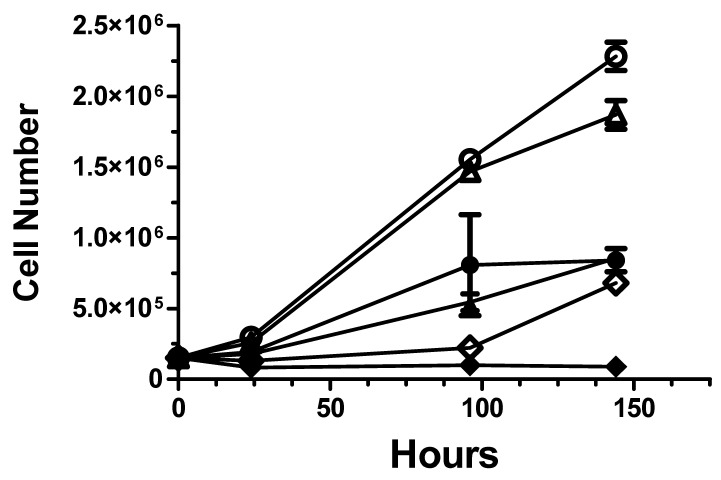
Opaganib sensitizes PAN02 cells to killing by radiation In vitro. Cells were plated and treated with 0 (open symbols) or 10 μM (filled symbols) opaganib and 0 (◯, ●), 5 (△, ▲) or 15 (◆, ◇) Gy of radiation. At the indicated times, viable cell numbers were counted using the trypan blue exclusion assay.

**In vitro effects of opaganib on cell radiosensitivity.** The effects of opaganib on radiation-induced killing of non-transformed murine IEC6 epithelial cells and murine PAN02 tumorigenic cells were analyzed in colony-forming and trypan blue cell counting assays. IEC6 or PAN02 cells were plated at low density and treated with Vehicle or 20 μM opaganib for 2 h prior to exposure to varying doses (0 Gy to 20 Gy) of radiation. For the non-transformed IEC6 cells, the IC_50_ and IC_90_ amounts of radiation in the absence of opaganib were 5.56 and 12.16 Gy, respectively. Addition of opaganib to the cultures increased the levels of radiation required to kill 50% and 90% of the IEC6 cells to 6.46 and 13.2 Gy, respectively (dose-modification factor = 1.16). Similarly, the IC_90_ for radiation alone increased from 12.2 Gy to 13.2 Gy (dose-modification factor = 1.08), suggesting that in this model of ‘normal’ tissue opaganib may provide protection from IR-induced cell death. In contrast, as shown in Figure 8, opaganib increased the killing of transformed PAN02 cells by radiation, particularly at the high dose of 15 Gy (*p* < 0.05). 

**Figure 9:**
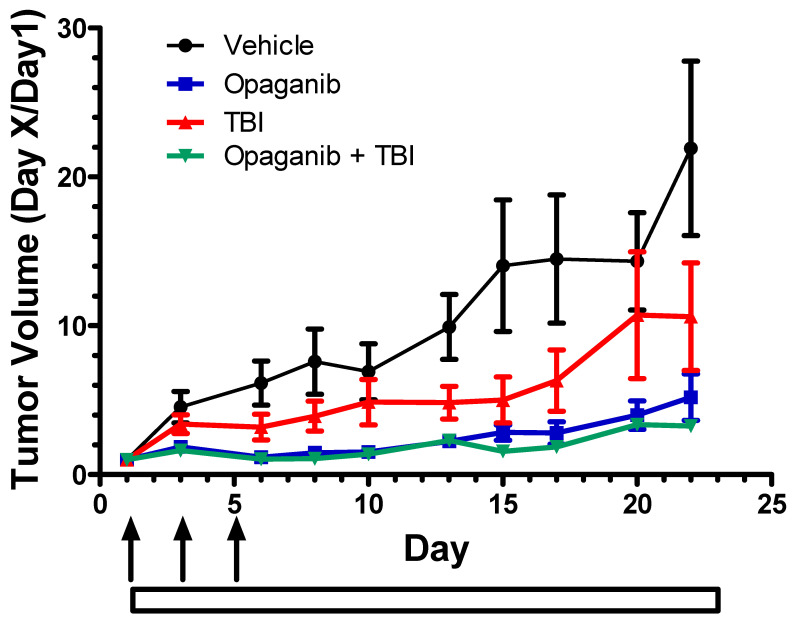
Antitumor efficacy of opaganib and radiation toward pancreatic tumors. C57/BL6 mice were subcutaneously injected with 10^6^ PAN02 cells. When tumors reached 100–150 mm^3^, animals were randomly assigned into one of four groups (*n* = 10/group): Vehicle (●); 25 mg/kg opaganib daily (5×/week) (∎); fractionated TBI (1 Gy, 3 times ↑) (▲); or combination of opaganib and TBI (▼). Mice in the combination group were treated with opaganib 2 h prior to radiation.

**In vivo effects of combination of opaganib with radiation on tumor growth.** To simulate cancer patients receiving low-dose fractionated radiation and to evaluate antitumor activity of opaganib in combination with radiation, C57/BL6 mice were subcutaneously injected with 10^6^ PAN02 cells suspended in PBS/Matrigel. When tumors reached 100–150 mm^3^, animals were randomly assigned into one of four groups (*n* = 10/group): Vehicle, oral 25 mg/kg/day opaganib (5×/week), 1 Gy of TBI three times in the first week for 3 Gy total TBI (70 cGy/min), or combination of opaganib and TBI. Mice in the combination group were treated with opaganib 2 h prior to radiation on each radiation day. Body weights were tracked to assess the overall health of the mice until sacrifice. Mice that received fractionated TBI alone had modest decreases in body weight (approximately 10%). Opaganib-treatment alone did not affect the average body weight, and opaganib did not prevent the decrease from radiation. As shown in Figure 9, control PAN02 tumors (vehicle only) grew at the fastest rate necessitating euthanasia after approximately 3 weeks. Treatment with either TBI alone or opaganib alone substantially reduced tumor growth (*p* < 0.05 and *p* < 0.001, respectively). Treatment with opaganib in combination with TBI resulted in significantly reduced tumor growth compared to the control group or to the TBI alone group (*p* < 0.01 for each comparison), but was not significantly different from opaganib alone because of the strong antitumor activity of the drug in this model. Importantly, treatment with opaganib clearly did not protect tumors from radiation treatment. Similar studies were conducted in C57BL/6 mice bearing syngeneic subcutaneous tumors of B16 melanoma or E0771 breast cancer cells (not shown). For each of these tumor models, opaganib plus TBI had equal or better antitumor activity than TBI alone; however, the strong antitumor activity of radiation alone in these models precluded statistical demonstration of further efficacy from the opaganib plus TBI combination. As with the PAN02 model, opaganib did not diminish the tumor response to fractionated radiation treatment, and did not increase weight loss from radiation treatment. 

**Figure 10:**
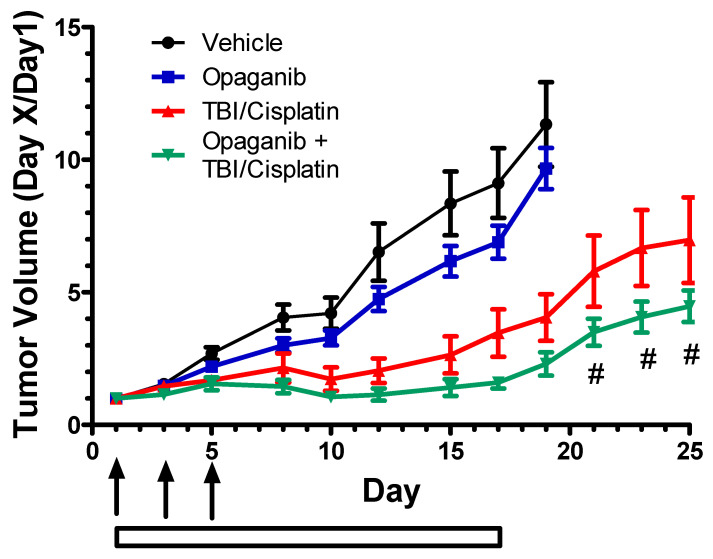
Antitumor efficacy of opaganib and chemoradiation toward Head and Neck SCC tumors. NCr nu/nu mice were injected subcutaneously with human FaDu tumor cells. When tumors reached 100–150 mm^3^, mice were randomized and treated with: Vehicle alone (●); opaganib 50 mg/kg/day, 5×/week (∎); fractionated TBI (3 Gy, 3 times ↑) plus cisplatin (2 mg/kg on all TBI days, 2 h pretreatment) (TBI + cisplatin) (▲); or TBI + cisplatin + opaganib (▼). Mice in the combination group were treated with opaganib 2 h prior to radiation. ^#^ indicates *p* < 0.02 for TBI + cisplatin + opaganib compared with TBI + cisplatin.

To assess the effects of opaganib when combined with current standard-of-care therapies for Head & Neck cancer (radiation + cisplatin), NCr nu/nu mice were injected subcutaneously with human FaDu squamous cell carcinoma cells. When tumors reached 100–150 mm^3^, mice were randomized and treated with: Vehicle alone; opaganib at 50 mg/kg/day, 5×/week; 9 Gy fractionated TBI + cisplatin; or TBI + cisplatin + opaganib. Mice in the combination group were treated with opaganib 2 h prior to radiation. As shown in Figure 10, treatment with opaganib alone slightly reduced tumor growth, while TBI + cisplatin substantially reduced tumor growth as compared to the control (vehicle) group (*p* < 0.001). Treatment with opaganib in combination with TBI + cisplatin provided the greatest reduction in tumor growth, and was significantly better than TBI + cisplatin on Day 21 and after (*p* < 0.02). As with the syngeneic tumor models, opaganib did not diminish the tumor response to fractionated radiation + cisplatin treatment, and did not increase weight loss from radiation + cisplatin treatment. 

## 3. Discussion 

Drugs capable of protecting against acute tissue damage (hematopoietic, germinal and epithelium of skin and gastrointestinal tract) and chronic pathologies (cancer, pulmonary fibrosis) resulting from exposure to IR are needed for adjunctive care during radiation therapy for cancer patients and also to safeguard military personnel, first responders and civilians from accidental or terroristic exposure to nuclear materials. No drug available today has all the qualities of an ideal radioprotector [48,49,50,51,52]. Amifostine, which is considered to be the “gold standard” in radioprotection [53,54,55,56], has been approved for limited clinical use in cancer patients undergoing intense, but local, head and neck radiotherapy to minimize salivary gland injury. However, amifostine is not well tolerated by either animals or humans when administered at doses that would need to be used to protect against TBI as opposed to localized cancer radiotherapy [53,57]. Therefore, there remains a significant need for improved systemic agents that protect against GI-ARS. 

Because of the accumulating evidence for the roles of sphingolipid metabolism in mediating the pathologies of a variety of inflammatory diseases, many studies have addressed the possibility of suppressing S1P formation as an innovative approach to therapy (reviewed in [16,17,18,19]). In particular, the actions of inflammatory cytokines are mediated by activation of S1P production by sphingosine kinases (SKs). For example, TNFα induces S1P production in endothelial cells [58,59], hepatocytes [60], neutrophils [61], monocytes [62], fibroblasts [63] and lung adenocarcimona cells [63] by activation of sphingomyelinase, ceramidase and SKs. Within endothelial cells, S1P activates NFκB thereby inducing the expression of multiple adhesion molecules and COX-2 resulting in PGE_2_ synthesis. Similarly, SKs have been demonstrated to regulate pro-inflammatory responses triggered by TNFα in primary human monocytes. S1P mimics the ability of TNFα to induce the expression of COX-2 and the synthesis of PGE_2_ in fibroblasts, and knock-down of SK by RNA interference blocks these responses to TNFα but not S1P [63]. S1P is also a mediator of Ca^2+^ influx during neutrophil activation by TNFα and other stimuli, leading to the production of superoxide and other toxic radicals [64,65]. 

Opaganib was originally developed to provide a new anticancer and anti-inflammatory drug; however, more recent demonstration that it has direct antiviral activity prompted clinical trials to assess its therapeutic activity against COVID-19 [46]. Because IR activates sphingolipid metabolism and this regulates both advantageous (tumor cell killing) and pathologic (local and systemic inflammation), we have now examined the effects of opaganib on responses to IR in vitro and in vivo. These multifactorial studies examined the ability of opaganib to prevent toxicity from IR following exposure of the entire body, i.e., a fully exposed hematopoietic system, or exposure with approximately 5% of the bone marrow shielded, which results in GI-ARS in the absence of hematologic ablation. Additionally, we sought to determine if opaganib can be effectively combined with radiotherapy for cancer patients.

In the initial studies, C57BL/6 mice were exposed to varying levels of TBI after pretreatment with orally administered opaganib. This was intended to model the prophylactic treatment of cancer patients scheduled for radiotherapy, military personnel engaged on a nuclear battlefield or first-responders to a nuclear event. Pretreatment with opaganib substantially reduced the lethality of TBI, shifting the LD_75_ by 2 Gy thereby allowing survival to an otherwise lethal radiation dose. Specifically, there was no survival of untreated mice following exposure to 9.5 Gy TBI; while 75% of mice that received a single oral dose of opaganib survived this radiation dose. Mechanistically, this protection was associated with excellent accumulation of opaganib in the small intestines, prevention of radiation-induced S1P and TNFα elevations, and reduced morphologic damage to this tissue. 

The efficacy of opaganib prophylaxis supported studies on the potential use of opaganib for the mitigation of radiation toxicity when given after radiation exposure which were supported by BARDA. In these studies, opaganib was administered 24 hs after the radiation exposure to mimic the scenario for treatment of civilian populations following radiation exposure from a terroristic or accidental nuclear event. Because it is expected that human exposure to radiation would not involve the entire body, a partially shielded model was used in all of these studies, in which the left hind leg of each mouse is shielded to protect approximately 5% of the bone marrow from the radiation. While these studies were underway, the US Department of Defense funded a supplement to the BARDA contract to assess the ability of opaganib to protect against GI-ARS when given 4 to 24 h prior to radiation exposure. 

In the partially shielded model, a single dose of opaganib given 4 h prior to IR provided greater protection than did administration of opaganib at earlier times. These results were generally expected considering the half-life of opaganib is approximately 4 h in mice [27]. Biodistribution analyses demonstrated that high levels of opaganib were present in the small intestine by 3 h after drug administration and were maintained for at least 7 h. Repeated dose studies demonstrated optimal protection when opaganib was given before and after radiation exposure. However, specific to the unintended radiation exposure paradigm (accidental or terroristic), repeated dose studies demonstrated that opaganib can be effective in providing radiation protection if started as late as 24 h after radiation. The efficacy demonstrated when given 24 h post exposure supports the development of opaganib for protection against GI-ARS following unintended radiation exposure. Specifically, strategic stockpiling of opaganib could provide a level of protection against radiation toxicity following a nuclear accident or a terrorist attack. Additionally, prophylactic treatment with opaganib could potentially benefit military or first-responder personnel at risk for radiation exposure.

**Figure 11:**
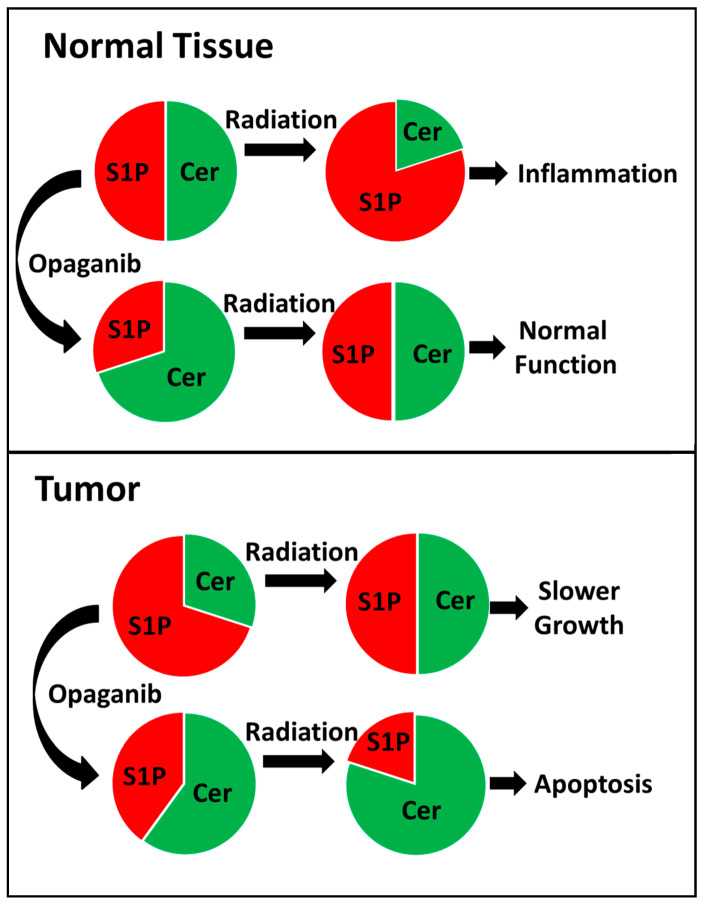
Model for prevention of GI-ARS and concurrent sensitization of tumor cells to killing by radiation. Levels of S1P and ceramides are represented by red and green slices, respectively. In normal GI tissue, radiation causes an increase in S1P leading to inflammatory tissue damage. Because opaganib treatment reduces the basal level of S1P in the GI tissue, the increase following radiation is insufficient to generate the pathologic inflammatory tissue response. In tumor tissue, the basal level of S1P is higher than normal tissue due to growth factor and/or oncogene driven sphingolipid hydrolysis, and in this case radiation elevates ceramide levels thereby decreasing tumor growth. Treatment of tumors with opaganib restores the resting S1P/ceramide balance and prevents radiation-stimulation of S1P formation, leading to elevation of ceramide sufficient to drive tumor cells into apoptosis.

Radiation toxicities, such as oral mucositis and radiation caries in Head & Neck cancer patients [66,67] and radiation enteritis in cancer patients receiving abdominal or thoracic radiation [68,69,70], are common and impact the patients’ quality of life. Additionally, toxicity to normal tissue may force reduction of radiotherapy dosage thereby impairing overall efficacy. The ability of opaganib to protect normal tissue from radiation damage supports its use in combination with radiotherapy for cancer patients. The current in vitro studies demonstrate that opaganib increases the toxicity of radiation toward tumorigenic cells, and this is consistent with reports by others that increased levels of ceramide (as occur in opaganib-treated tumor cells) sensitizes tumor cells to killing by radiation (reviewed in [26,71]). Importantly, opaganib administration to non-transformed cells (IEC6) did not increase their radiation sensitivity. The in vitro results were extended by several in vivo tumor models for confirmation that opaganib does not diminish the tumor response to or increase toxicity from fractionated radiation treatment. In the chemoradiation model of Head & Neck cancer (FaDu cell xenografts), the combination of radiation + cisplatin + opaganib improved antitumor activity over radiation + cisplatin. Taken together, these in vitro and in vivo studies support the hypothesis that opaganib will be beneficial to cancer patients by protecting the normal tissue from radiation toxicity, while concomitantly enhancing the radiation sensitivities of tumor cells, thereby resulting in increased efficacy of cancer radiotherapy (Figure 11). In addition to patients with Head & Neck cancer, opaganib treatment may benefit other cancer patients treated with abdominal and/or pelvic radiation. 

In parallel, the current data demonstrate that opaganib protects against radiation toxicity across a wide range of exposure scenarios, and that the drug can be effectively combined with cancer radiotherapy. Radioprotection is manifested as decreased mortality and GI damage in mice treated with opaganib and exposed to total-body or partially shielded IR, while improved radiotherapy is indicated by enhanced antitumor activity in the absence of increased toxicity to fractionated IR. The studies further demonstrate that the optimal effects of opaganib are provided when the drug is given prior to radiation exposure and for several days following radiation exposure. This is entirely feasible for the use of opaganib in combination with radiotherapy for cancer patients, as well as for the use of the drug as a preventative agent when the risk of radiation exposure exists. However, opaganib treatment did provide substantial survival benefit even when its administration was delayed until after radiation exposure, making it a potentially useful drug for mitigation of toxicity following unintended exposure to radiation. Opaganib has reached a high Technology Readiness Level (as defined by BARDA) reflected by the established safety of opaganib in patients with cancer or COVID-19, the known stability of the drug product, and the ease of oral administration, and this supports further development of opaganib as MCM in the BARDA program for emergency preparedness against radiation threats.

## 4. Materials and Methods

**Materials.** Clinical grade opaganib synthesized by ChemPacific was used for all studies. PAN02, FaDu, B16, E0771 and IEC6 cells were purchased from the American Type Culture Collection. 

**Total body irradiation.** Six-to-eight week old male C57BL/6 mice weighing 20 to 24 g were obtained from Jackson Laboratories (Bar Harbor, ME, USA) and acclimated for at least 3 days before experimentation. All the mice were housed four per cage, and received food and water ad libitum. Mice were exposed to ionizing radiation on a rotating platform using a JL Shepherd Model 143 ^137^Cesium γ-irradiator (JL Shepherd, Glendale, CA, USA) at a dose rate of 2.6 Gy/min, and then monitored for up to 30 days.

**Irradiation with partial shielding.** Six-to-eight week old male C57BL/6 mice weighing 21 to 29 g were obtained from the Jackson Laboratories and acclimated for at least 13 days before experimentation. All the mice were housed individually and received food and water ad libitum. Mice were irradiated in groups up to 12 in a custom designed restrainer in which their left pelvic limb is extended and maintained in position with an elastic band. The left pelvic limb was shielded with a cerrobend structure to provide an estimated 5% bone marrow shielding. Mice were exposed to 60 cGy per minute from a ^60^Co gamma source. Doses of 15.25 and 16.0 Gy were used in several experiments because they were determined to result in approximately LD_30_ and LD_70_ in vehicle-treated mice using this irradiation setup. In the single drug dose experiments, treatment groups consisted of 0 (vehicle), 100 or 300 mg/kg opaganib given at −24, −4, +4 or +24 h in respect to irradiation. In the multiple dose experiments, the −4 h dosing regimens consisted of 0 (vehicle), 50 or 100 mg/kg opaganib given at −4, +4 h and then BID for 3 days post irradiation, or 300 mg/kg opaganib given at −4 and +4 h followed by BID dosing with either 50 or 100 mg/kg opaganib. The +4 h dosing regimens consisted of either 50 or 100 mg/kg opaganib given at +4 h and then BID for 3 days post irradiation. The +24 h regimen consisted of 50 or 100 mg/kg opaganib given at +24 h BID and then BID for 3 days post irradiation.

**Quantification of opaganib in small intestine.** At the indicated times after oral opaganib administration, the mice were euthanized and the small intestines were isolated and homogenized in lysis buffer containing 1% Nonidet *p*-40, 50 mM HEPES pH 7.4, 500 mM NaCl, and 1% protease inhibitor cocktails (Sigma-Aldrich, St. Louis, MO, USA). The homogenates were centrifuged at 20,000 rpm at 4 °C for 10 min, and the supernatants were collected. Supernatants were extracted twice with ethyl acetate, and the extracts were dried under nitrogen at 35 °C. The samples were then analyzed by reverse-phase HPLC on a Supelco discovery C18 column (20′2.1 mm) with the mobile phase consisting of methanol in 0.1% formic acid as solvent A and water with 5% acetonitrile in 0.1% formic acid as solvent B. The gradient started with 30% solvent A: 70% Solvent B, which was linearly increased to 100% Solvent A over 9 min. Absorbance at 254 nm was monitored and quantification was achieved by comparing the sample peak area with those of the pure opaganib.

**ELISA assays for cytokines and S1P.** The small intestines were processed as indicated above, and immunoreactive TNFα levels were quantitated using ELISA kits specific for mouse TNFα (Thermo Scientific, Hanover Park, IL, USA). The same preparations were used to quantify S1P levels using ELISA kits (Echelon Biosciences, Salt Lake City, UT, USA). Levels of IL-1β and IL-6 in the lysates were determined by Luminex assays performed by the Cytokine Core Laboratory at the University of Maryland, Baltimore.

**Histology of intestines.** Following sacrifice, small intestines were collected and the intestinal contents were removed. The small intestines were fixed with 4% paraformaldehyde and embedded in paraffin, and thin sections were stained with hematoxylin and eosin (H&E). For villi cellularity determinations, three sections from each mouse were counted and averaged. Histology scores were determined by assessing inflammation severity, inflammation extent and crypt damage multiplied by percent area involvement. Six regions were evaluated per slide and averaged to produce a final score for that mouse.

**Radiosensitivity of normal and cancer cells in vitro.** IEC6 cells were plated at low density and treated with vehicle or 20 μM opaganib 2 h prior to exposure to varying doses (0 Gy to 20 Gy) of IR. Following radiation, cells were incubated for 2 weeks with media and original opaganib concentration refreshed every 4 days. Cell colonies were fixed with methanol, stained with crystal violet and colonies with 50 or more cells were counted. Because PAN02 cells did not form discrete colonies, these cells were analyzed using trypan blue exclusion assays to quantify cell proliferation. PAN02 cells were plated at 10^5^ cells/well and treated with opaganib for 2 h before exposure to radiation doses of 0, 5 or 15 Gy. Cells were collected at 24, 96 or 144 h after radiation and total viable cell numbers were determined using the trypan blue exclusion assay. 

**Tumor studies.** In the syngeneic tumor model, C57/BL6 mice were subcutaneously injected with 10^6^ PAN02 tumor cells suspended in PBS/Matrigel. When tumors reached 100–150 mm^3^, animals were randomized into four groups (*n* = 10/group): Vehicle (46% PEG400: 47% Saline: 7% ethanol), 25 mg/kg opaganib daily (5×/week), 1 Gy TBI (70 cGy/min) three times/week or combination of opaganib and TBI. Mice in the combination group received opaganib 2 h before IR on each radiation day. In the Head & Neck tumor model, NCr nu/nu mice (NCI) were injected subcutaneously with human FaDu tumor cells. When tumors reached 100–150 mm^3^, mice were randomized and treated with: Vehicle alone; opaganib 50 mg/kg/day, 5×/week; fractionated radiation (3 Gy 3×/week) plus cisplatin (2 mg/kg on all IR days, 2 h pretreatment) (IR + cispt); or IR + cispt + opaganib. Mice in the combination group were treated with opaganib 2 h prior to IR on each radiation day.

**Statistics.** Mouse survival rates following IR were compared using the Kaplan–Meier approach with the Gehan-Breslow-Wilcoxon test using GraphPad Prism 5 software. Other data were analyzed by one-way ANOVA using the Tukey post hoc test. Differences were considered to be statistically significant when *p* < 0.05. Error bars in figures represent the mean ± standard deviation of the treatment groups calculated with GraphPad Prism 5.

## Data Availability

Not applicable.

## IACUC Statement

The animal study protocols were approved by the Institutional Animal Care and Use Committee (IACUC) of the Penn State University College of Medicine (Protocol number 201347170) in compliance with U.S. Government Principles for the Utilization and Care of Vertebrate Animals Used in Testing, Research and Training, which complies with the PHS Policy on Humane Care and Use of Laboratory Animals, and the United States Department of Agriculture Animal Welfare Act guidelines, and CiToxLab NorthAmerica in accordance with the principles outlined in the current Guidelines published by the Canadian Council on Animal Care and the Guide for the Care and Use of Laboratory Animals.
